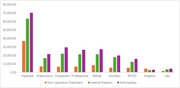# Comparing overall medical costs of operative versus nonoperative treatment for femoral neck fractures among Alzheimer’s disease patients: a retrospective cohort study

**DOI:** 10.1002/alz.085439

**Published:** 2025-01-09

**Authors:** Yijiong Yang

**Affiliations:** ^1^ Florida State University, Tallahassee, FL USA

## Abstract

**Background:**

Addressing femoral neck fractures resulting from ground‐level falls in older adults with Alzheimer’s disease (AD) involves a personalized treatment plan, leading to a substantial economic burden on the healthcare system. The debate surrounding the advantages and disadvantages of surgical interventions versus non‐operative approaches for femoral neck fractures in older individuals with AD remains a topic of active discussion.

**Method:**

In this retrospective cohort study, the total medical expenses associated with operative and non‐operative therapies were compared while adjusting for patients' demographics and baseline health conditions. The study included Optum beneficiaries diagnosed with Alzheimer’s disease (AD) who filed an initial claim for femoral neck fracture between January 1, 2012, and December 31, 2017. Generalized linear regression with a gamma distribution and log link was performed to compare the adjusted overall medical costs between treatment groups controlling for patients’ demographic characteristics (age, gender, race, residential region), insurance type, and baseline health status.

**Result:**

A total of 4,157 AD patients with femoral neck fracture was identified. This included 1,508 cases undergoing internal fixation, 2,334 cases of arthroplasty surgery, and 315 cases of non‐operative treatment. Among them, 59.8% were female (n = 2,487), and the median age was 81 years. The adjusted average total medical costs for arthroplasty and internal fixation were $207,392 and $170,210, which were higher than average total cost for non‐operative group (mean = $63,041). Arthroplasty treatments exhibited overall medical costs 2.56 times higher (95% CI: 2.288 – 2.875) than those of non‐operative treatment, while internal fixation’s overall costs were 2.33 times higher (95% CI: 2.067 – 2.628) than non‐operative treatment. Comorbidities such as history of fall, sarcopenia/muscle weakness, abnormal weight loss, depression, and fatigue had a significant influence on the overall medical cost. Patients with history of fall had an estimated 1.22 times higher medical cost compared with those without history of fall.

**Conclusion:**

The operative treatment groups exhibited higher overall medical costs. The overall costs encompassed not only the direct medical costs associated with surgery but also post‐surgery rehabilitation expenses in nursing homes or home health settings.